# Design of New Dispersants Using Machine Learning and Visual Analytics

**DOI:** 10.3390/polym15051324

**Published:** 2023-03-06

**Authors:** María Jimena Martínez, Roi Naveiro, Axel J. Soto, Pablo Talavante, Shin-Ho Kim Lee, Ramón Gómez Arrayas, Mario Franco, Pablo Mauleón, Héctor Lozano Ordóñez, Guillermo Revilla López, Marco Bernabei, Nuria E. Campillo, Ignacio Ponzoni

**Affiliations:** 1 ISISTAN (CONICET-UNCPBA) Campus Universitario—Paraje Arroyo Seco, Tandil 7000, Argentina; 2 Institute of Mathematical Sciences (ICMAT-CSIC), Nicolás Cabrera, nº 13-15, Campus de Cantoblanco, UAM, 28049 Madrid, Spain; 3 AItenea Biotech, Parque Científico de Madrid, Ciudad Universitaria de Cantoblanco, Calle Faraday, 7, 28049 Madrid, Spain; 4 Campus Pirineos, CUNEF Universidad, Calle de los Pirineos, 55, 28040 Madrid, Spain; 5 Institute for Computer Science and Engineering (UNS–CONICET), San Andrés 800, Campus Palihue, Bahía Blanca 8000, Argentina; 6 Department of Computer Science and Engineering, Universidad Nacional del Sur, San Andrés 800, Campus Palihue, Bahía Blanca 8000, Argentina; 7 Department of Organic Chemistry, Institute for Advanced Research in Chemical Sciences (IAdChem) UAM, 28049 Madrid, Spain; 8 Repsol Technology Lab DC Technology & Corporate Venturing, Agustín de Betancourt s/n, Móstoles, 28935 Madrid, Spain; 9 CIB Margarita Salas (CSIC), Ramiro de Maeztu, 9, 28740 Madrid, Spain

**Keywords:** polyisobutylene, blotter spot, artificial intelligence, Bayesian regression

## Abstract

Artificial intelligence (AI) is an emerging technology that is revolutionizing the discovery of new materials. One key application of AI is virtual screening of chemical libraries, which enables the accelerated discovery of materials with desired properties. In this study, we developed computational models to predict the dispersancy efficiency of oil and lubricant additives, a critical property in their design that can be estimated through a quantity named blotter spot. We propose a comprehensive approach that combines machine learning techniques with visual analytics strategies in an interactive tool that supports domain experts’ decision-making. We evaluated the proposed models quantitatively and illustrated their benefits through a case study. Specifically, we analyzed a series of virtual polyisobutylene succinimide (PIBSI) molecules derived from a known reference substrate. Our best-performing probabilistic model was Bayesian Additive Regression Trees (BART), which achieved a mean absolute error of 5.50±0.34 and a root mean square error of 7.56±0.47, as estimated through 5-fold cross-validation. To facilitate future research, we have made the dataset, including the potential dispersants used for modeling, publicly available. Our approach can help accelerate the discovery of new oil and lubricant additives, and our interactive tool can aid domain experts in making informed decisions based on blotter spot and other key properties.

## 1. Introduction

Formulated lubricants are essential components for countless industrial and mobility-related activities, and they represent an over $60 billion global chemical enterprise [1,2,3]. Regardless of the application, approximately 80–95 wt% of the lubricant’s total weight composition is the lubricating base oil, which can be either petroleum-based or synthetic in nature, such as poly-alpha-olefins or synthetic esters. In addition to the base oil, a formulated lubricant incorporates in the formula a series of additives, e.g., an additive package, which improves the physicochemical properties, thus providing unique essential characteristics depending on the specific application. The United Nations’ global goal of net zero emissions by 2050 [4,5,6] is demanding an increase in the production of high-performance fuels and lubricants from sustainable feedstock in order to reduce dependence on petroleum-based products and mitigate the environmental footprint. In addition, additives have to be taken into consideration because of their contribution to the performance of a lubricant. Additives’ sustainability needs to be evaluated not only with respect to their manufacturing process but also regarding how they improve the performance and durability of the formulated lubricant. Considering the small time-window available to achieve the net-zero emission goal, the design of a new generation of lubricant additives that can improve sustainability by helping with fuel economy, reducing carbon dioxide emissions and prolonging the useful life of lubricants, can benefit from the speed-up provided by an artificial intelligence (AI) based approach to molecular design.

Dispersant additives, which have been extensively used since the 1950s, are one of the main components of the total additive percentage of a formulated lubricant for modern internal combustion engines. Under the harsh operating conditions of an engine, soot is produced as a result of incomplete oxidation/combustion of the fuel. In an engine, soot aggregation increases lubricant viscosity, preventing regular flow, causing corrosion, deposit formation and mechanical wear. Dispersants are polymeric surfactant-like amphiphilic molecules characterized by at least one hydrophobic, oil soluble ‘tail’ polymer backbone component, often polyisobutylene (PIB), and at least one hydrophilic, polar ‘head’ unit that adsorbs onto the surface of ultrafine carbon deposit precursors (mainly sludge soot particles) stabilizing them by acid–base or electrostatic interactions, thereby reducing their aggregation [7,8,9,10]. An efficient dispersant design requires tailoring the nature of the chemical interactions to meet the performance characteristics of a particular engine, for which a number of parameters need to be fine-tuned [11,12]. Despite the knowledge available, the chemistry for production of dispersants in use today remains limited. Therefore, there are significant opportunities for innovation. Polyisobutylene succinimide (PIBSI)-based dispersants, initially developed in 1965 by Le Suer and Stuart, are still the most common ashless dispersants used in engine oil [13,14]. They are composed of a polyamine polar head, which is connected to a long non-polar polyisobutylene stabilizing tail by a succinimide group.

The design of dispersants involves a deep understanding of which molecular architecture can best provide the functions required for the target application while considering the potential interactions of amine and imide functional groups with other additives. This task is typically carried out through trial and error, coupled with chemical intuition, but the process is expensive and time-consuming. In sharp contrast, artificial intelligence (AI) holds great potential to guide the design of next generation materials because of its capability for solving multiobjective optimization problems, thus allowing economic and time savings. In this sense, AI-based approaches are increasingly used in the design of complex materials [2,3,15]. In particular, several subproblems associated with the design of lubricants [16,17,18] and fuels [19,20] have been addressed using machine learning techniques. More specifically, quantitative structure–activity relationships (QSAR) modeling that has historically been applied to computer-aided drug design can be adapted to process data from different domains, in particular, materials design [21,22]. QSAR models establish linear or nonlinear relationships between descriptor values that are computed from molecular structures and experimentally measured physicochemical or biological activity properties. Then, these QSAR models are used to design new chemical structures with the desired properties. However, the use of these methodologies in the design of new dispersants is still incipient. In Menon et al. [23], a computational method for understanding and optimizing the properties of complex physical systems is presented. This method is known as Hierarchical Machine Learning (HML), which combines physical and statistical modeling. The first implementation of HML embedded domain knowledge into polymer dispersants to probe their effect in a cement-based system, and it constitutes one of the few prior studies that we have identified on the use of machine learning for the study of dispersants. Nonetheless, it is important to mention that the blotter spot property, modeled in our present work, is not addressed by Menon et al. Furthermore, their methodology does not incorporate a visual analytics approach for expert users to visually evaluate potential molecules. Finally, Jablonka et al. [15] presented an active learning algorithm for de novo polymer design focused on simulation of dispersancy characteristics. This work showcases how simulation and machine learning techniques can be coupled to discover materials. However, we would like to clarify that the goals of this work differ from the present paper. While they proposed a de novo design strategy using molecular simulations, we do not propose a generative model method. Instead, our approach is focused on evaluating and prioritizing molecules designed by human experts based on fundamental principles.

In this context, the primary objective of our research is to aid the design of efficient dispersants through AI-based modeling and interactive visualizations, leading to reduced operational expenses and faster discovery of novel dispersants. Our hypothesis is that the polar head is mainly responsible for dispersant activity by absorbing onto the surface of soot particles. In this sense, a question that arises is whether a computational approach, based on machine learning and visual analytics, to determine the relationship between the chemical structure of the polar head and a given dispersancy measure is suitable to guide virtual screening in the design of new materials with an expected dispersancy profile. In particular, in this paper, we describe a machine learning based tool for dispersant design and optimization, which is focused on the prediction of a key performance property: dispersancy efficiency (measured using the blotter spot test) based on the molecular structure of the polar head of molecules. Complementary strategies were developed based on a combination of unsupervised and supervised learning, nonlinear dimensionality reduction methods and data visualization strategies. The resulting inol was statistically validated, and its application is illustrated by the evaluation of a series of virtual PIBSI molecules derived from a known reference substrate, by testing the potential of the proposed tool in virtual screening problems.

## 2. Materials and Methods

In this section, we present all key issues related to the data curation, computational representation of molecules, proposed predictive modeling and design of the visual analytics tool. In this sense, two computational models were proposed as part of our tool in order to provide different perspectives for predicting the dispersancy efficiency of molecules: a structural similarity-based model and a Bayesian regression model. Thus, the best candidates for dispersants can be selected by the expert’s assessment of molecules with the highest prediction values from each model, taking into account predictive uncertainty.

### 2.1. Dataset of Potential Dispersants: Data Compilation and Curation

The first step of our work was to compile a dataset from the literature with 83 potential dispersants, mostly PIB-succinimide dispersants (PIBSIs), by conducting a manually curated data extraction process from an extensive collection of articles [7,8,24,25,26,27,28,29,30,31,32,33,34,35,36,37]. The dispersant capacity of the candidate molecules, namely dispersancy efficiency, was selected as the property for prediction and optimization purposes. Among the standard tests for measuring the merit of dispersancy of engine oils, the blotter spot test (ASTM D7899) was selected. This test is one of the oldest techniques, and provides an excellent assessment of the lubricant’s dispersancy performance of engine oils where pollutants of diverse sources such as soot from combustion, metal particles from wear or insoluble products resulting from the oxidation of the oil may contaminate the lubricant.

Additionally, results from dispersancy evaluation methods based on thermal oxidative decomposition procedures were processed into a parameter known as reduction factor [24,25,35,38] (i.e., *R-Factor = soot reduction ratio* × *initial soot weight / concentration of dispersant*). This allowed us to normalize the performance of dispersants as a common arbitrary unit from different experimental techniques. As a result of the incorporation of the R-factor calculation, the final dataset includes molecular structures with known blotter spot and/or R-factor values. However, as we explain below, the experiments presented in this work were focused on blotter spot values, since it is the most predominant property available for the chemical structures included in the dataset.

The complete dataset of 83 molecular structures was used for the two-dimensional visualization based on structural similarity. For model validation purposes, we keep the 53 potential dispersants for which blotter spot values are available and paired them with the chemical structure of the polar head of the molecule. In view of the challenge presented by the sparse data found in the literature, we had to adapt our modeling approach to work in this regime. The scarcity of labeled data prevents us from using deep learning based approaches [39,40]. Instead, Bayesian regression models combined with visual analytic strategies were used, as they have proven to be beneficial in small data regimes [41,42]. The collected data have been made publicly available (see Data Availability section).

### 2.2. Computational Representations of Molecules

In our dataset of potential dispersants extracted from the literature, each molecule is represented by the SMILES code [43] corresponding to its polar head only. Then, classical molecular descriptors were computed using Mordred (version 1.2.0) [44]. This tool allows the calculation of 1613 molecular descriptors from two-dimensional structures and 213 from three-dimensional structures. Most of these descriptors represent topological properties calculated traversing the molecular graph underlying the chemical structure of each compound, where nodes represent atoms and edges represent chemical bonds.

Each molecule is thus represented by a vector of numerical values corresponding to different descriptors. This allows a tabular representation of the dataset.

Additionally, SMILES-based molecular embeddings calculated from a pre-trained model based on recurrent neural networks (RNN) with long-short term memory cells [45] were also obtained for each potential dispersant. The RNN-based model was trained with millions of molecules in the tasks of “prediction of the next token” and “prediction of masked tokens”, where in a chemical scenario, a token represents part of the SMILES code of the molecule [46]. Therefore, latent representations of the molecules (embeddings) that are expected to capture relevant information contained in the SMILES codes are obtained using a pre-trained model [47].

### 2.3. Model Based on Structural Similarity

Our first modeling strategy aims to predict the dispersancy efficiency of new molecules based on structural similarity with existing molecules with known dispersancy efficiency values. The hypothesis behind this strategy is underpinned by the QSAR modeling principle, which indicates that structurally similar compounds should exhibit similar properties [21]. In our work, we employed molecular descriptors as numerical values to characterize the chemical structures, capturing the compounds’ topological information and atom properties. Therefore, in this context, structural similarity refers to the similarity of the molecular descriptors and not the overall similarity of the chemical structures. In this regard, a distance calculation is performed on the metric space defined by the values of the molecular descriptors that were previously identified as relevant to characterize the dispersancy efficiency of a compound. For this reason, the selection of the descriptors used to define this type of model plays a central role. Furthermore, as it will be explained in detail in Section 2.6, this multidimensional metric space is projected into a two-dimensional space to allow a more intuitive visual exploration of the model and its predictions.

#### 2.3.1. Molecular Descriptor Selection

For this first model, we focused on classical molecular descriptors. These descriptors refer to specific molecular properties, thus facilitating their interpretation and enabling human experts’ intervention during the analysis and visual exploration of the results of the model. In particular, we focus on 2D molecular descriptors only. Then, descriptors with undefined values, constant values and those correlated with a value greater than 0.95 were discarded. A final set of 425 molecular descriptors was obtained. Then, the first step for designing the similarity-based model was to conduct a feature selection procedure to identify a reduced subset of molecular descriptors statistically related to the target property. This subset defines the underlying metric space used by the model.

The relevance of the selected descriptors in the model was established through a two-stage process. The first stage aims at pre-selecting different subsets of molecular descriptors through fully automated strategies, using machine learning methods, and also through a selection made by experts in the chemical domain. The second stage aims to compare the pre-selected subsets of descriptors through an exploratory visual analysis of the relationships among them, following a human-in-the-loop methodology [48]. The decision to carry out the selection of descriptors in two stages for this model is due to the need of obtaining a small subset of descriptors. This is because our visual-oriented similarity-based model that we later use to assess candidate molecules (Section 2.6) requires condensing the visualizations to a human-scale that facilitates the analysis and interpretation of the molecules in terms of few descriptors. This reason will become even more apparent when the layout of the visualizations of the model is presented.

In the first stage, four pre-selected subsets of descriptors were obtained, three of them generated by applying descriptor selection methods based on machine learning techniques and the fourth one by using expert criteria. In this regard, the three subsets obtained through machine learning were the result of experimenting with Random Forests [49] and Decision Trees [50] in a repeated manner with different seed values. Decision trees are widely used as feature selection methods. Conceptually, based on the most information-rich features, a binary division procedure is followed where each bifurcation divides the chemical space into areas of similar target values. Random Forest is an ensemble of decision trees, where each tree is trained with a random subset of features and randomly sampled data instances. Both tree-based techniques calculate a score for each feature during the learning process. Then, the most important features can be easily ranked.

From different runs with Random Forest and Decision Trees, we selected the three subsets of descriptors that obtained the lowest error values. In each experiment, we kept the top-8 performing descriptors. Regarding the subset obtained through expert criteria, this was defined by an organic chemist who participated in the project. They identified key features for the design of this type of chemical structures that are also highlighted elsewhere in the scientific literature, such as polarity, orbital electronegativity, basicity and percent nitrogen composition. Keeping these guidelines in mind, five molecular descriptors related to these characteristics were selected. The detailed list with the pre-selected subsets of descriptors is summarized in Table 1.

In the second stage, these subsets were used as input to make the final selection of descriptors using ViDeAn [51]. Since several subsets of candidate molecular descriptors were obtained, this visual analytics tool allows for selecting descriptors following an expert-in-the-loop strategy. ViDeAn offers different coordinated views for visual interactive exploration of the relationships among molecular descriptors, the target property and candidate descriptor subsets based on different statistical metrics. In this way, the domain expert’s knowledge can be added to analyze the subsets of descriptors previously obtained by automated feature selection methods. Using the latter tool and with the help of the chemical expert, it was possible to visually explore relationships among molecular descriptors in terms of mutual information, linear correlation and the co-occurrence that they present in the pre-selected subsets. Furthermore, the individual relationship of each descriptor with the target property as well as the predictive capacity estimation of a subset of descriptors defined by the expert during data exploration was analyzed. As a result of this exploratory analysis, the final selection of descriptors was made up of AATS5d, ATSC5v and PEOE_VSA9.

AATS5d is the Moreau–Broto descriptor that measures the autocorrelation in the topological structure, at topological distance 5, of the atomic property linked to the sigma electrons of the atoms. ATSC5v is the Moreau–Broto descriptor that measures the autocorrelation in the topological structure, also for topological distance 5, of the atomic property linked to the volume of van der Waals atoms. Finally, the PEOE_VSA9 descriptor is computed as the sum of the van der Waals surface areas of the atoms whose PEOE partial charge (partial equalization of orbital electronegativities) is in the range of [0.05, 0.10). More information on how these descriptors are calculated is available in Todeschini and Consonni [52]. The three selected descriptors are used by our tool to analyze the structural similarity among the compounds of our dataset and to visually project those molecules in a two-dimensional space as it will be explained in detail in Section 2.6.

#### 2.3.2. Evaluation of New Molecules

In order to evaluate potential candidate molecules during a virtual screening study, a model based on structural similarity was carried out using a regression algorithm based on k-nearest neighbors [53]. This method is applied on the descriptor space to identify the most similar molecular structures in the dataset to the unseen candidate compound, and hence infer its dispersancy value. The predicted value is obtained by local interpolation of the associated target property values of the nearest neighbors. Then, the estimator of Equation (1) was defined to predict a target value for a new candidate compound *x* from its neighbors:

(1) $$\mathrm{WM}\left(x\right)=\sum_{i=1}^{k}\left(wcoef_{i}\cdot y_{i}\right),\;\mathrm{where}\;wcoef_{i}=\frac{\left(\sum_{j=1}^{k}d_{j}\right)-d_{i}}{2\sum_{j=1}^{k}d_{j}},$$

where *k* is the number of neighbors to consider, $y_{i}$ is the target property for each neighbor *i*, and $d_{i}$ is the distance between candidate *x* and neighbor *i*. The weight $wcoef_{i}$ is inversely proportional to the distance between the candidate compound and the neighbor *i*. The Weighted Mean (WM(x)) is the estimated property value for *x* calculated as the weighted average value of the target property of its neighbors.

A cross-validation procedure was carried out for the model evaluation. For this, we use two clustering techniques for assigning each molecular structure to a given data fold: KMeans and Density Based Spatial Clustering of Applications with Noise (DBSCAN) [54]. KMeans is one of the most commonly used clustering techniques. The process consists of randomly defining a number of *k* centroids (clusters) to seed the clustering process. Then, the molecules are assigned to one of those *k* groups taking into account a distance measure to the centroid. DBSCAN is one of the most widely used density-based clustering algorithm. DBSCAN does not require setting the number of clusters in advance, but rather the algorithm finds an optimal number of clusters based on an estimated density distribution of the data. The algorithm requires two important parameters: the distance threshold to consider two points as neighbors, and the minimum number of points for a region to be considered as a cluster. As per the sampling for each fold, a specific strategy was followed to ensure that structurally similar compounds are allocated evenly to different partitions. These clustering techniques were applied using the Python package sklearn.cluster: KMeans and DBSCAN [55,56]. For KMeans, a value of k = 4 was used. For DBSCAN, the parameters ϵ = 0.6 and min_samples = 3 were used for the threshold and the minimum number of samples per cluster. Once the similar compounds are grouped together, the next step consisted of distributing all the compounds belonging to a cluster in the different partitions until all the compounds in the dataset were assigned.

### 2.4. Bayesian Regression Model

We opted for a Bayesian approach as a second predictive modeling technique, given their ability to produce uncertainty estimates for their predictions, a key aspect in small data regimes [41]. In particular, we used the Bayesian Additive Regression Trees (BART) [57] model. BART is a Bayesian non-parametric model in which the unknown regression function is modeled as a sum of decision trees in which each tree is constrained, through a regularization prior, to be a weak learner.

In the context of regression analysis, Bayesian Additive Regression Trees aim to infer the unknown, nonlinear functional relationship, denoted by *f*, between a *p*-dimensional covariate vector *x* (representing molecular descriptors in the present case) and the output of interest *y* (represented by the blotter spot value). The probabilistic framework for this inference is expressed as

y=f(x)+ϵ

where the error term ϵ is assumed to be normally distributed with mean 0 and variance $\sigma^{2}$, i.e., $\epsilon ∼N(0,\sigma^{2})$.

BART approximates f(x)=E(y|x) through a sum-of-trees model $\sum_{j=1}^{m}g\left(x;T_{j},M_{j}\right)$. The formulation of this model begins with the specification of notation for an individual tree model. The model for the *j*-th decision tree comprises a binary tree $T_{j}$ that encompasses a set of interior node decision rules and a set of *b* terminal nodes with associated parameters $M_{j}=\left\{\mu_{1j},\mu_{2j},\ldots ,\mu_{bj}\right\}$. The decision rules are binary splits of the predictor space of the form {x∈A} vs. {x∉A} where A is usually a subset of the range of one of the dimensions of *x*. The binary tree is designed such that each instance *x* is linked to a single terminal node through the sequential application of decision rules. The function $g(x;T_{j},M_{j})$ assigns to *x* the corresponding $\mu_{ij}$ value from $M_{j}$. The sum-of-trees model can be concisely expressed as:

$$y=\sum_{j=1}^{m}g\left(x;T_{j},M_{j}\right)+\epsilon \;\epsilon ∼N\left(0,\sigma^{2}\right)$$

The BART model prevents overfitting by applying a regularization prior on the model parameters $\left(T_{1},M_{1}\right),\left(T_{2},M_{2}\right),\ldots ,\left(T_{m},M_{m}\right)$ and $\sigma^{2}$, ensuring that each decision tree is a weak learner. This completes the model specification. Further details on the prior specification can be found in Chipman et al. [57].

Given a set $D={\left\{\left(x_{i},y_{i}\right)\right\}}_{i=1}^{N}$ of *N* molecules with molecular descriptors $x_{i}$ and known blotter spot $y_{i}$ represented, the Bayesian setup induces a posterior distribution over the model parameters $p[\left(T_{1},M_{1}\right),\left(T_{2},M_{2}\right),\ldots ,\left(T_{m},M_{m}\right),\sigma^{2}|D]$. Samples from this posterior are generated using Bayesian backfitting Markov Chain Monte Carlo.

Finally, given a new unseen molecule with associated molecular descriptors *z*, samples from its posterior predictive blotter spot distribution can be easily obtained passing *z* through each of the generated posterior groups of *m* trees. The posterior predictive samples can be utilized to make point predictions and estimate the uncertainty of the blotter spot for the new molecule.

One of the notable advantages of the BART method is the capability to produce uncertainty estimates for the predictions, which is a significant departure from non-probabilistic methods such as Random Forest or Gradient Boosting. In our context, these uncertainty estimates are helpful to identify potential candidates for synthesis and are crucial in our small data regime. In particular, in an active learning setting, having estimates of uncertainty in the predictions made by the model allows for building a policy for synthesizing new targets that trades off between exploration and exploitation.

For training this model, all the 2D molecular descriptors and molecular embeddings were used. Features that were constant over the dataset, and those correlated with a value greater than 0.95 were discarded. Finally, a set of 939 features, including both molecular descriptors and embeddings, was obtained. The accuracy of various supervised learning models, both Bayesian and non-Bayesian, was compared to ensure that the Bayesian Additive Regression Trees perform similarly to state-of-the-art models in terms of predictive accuracy. As the number of features is much higher than the number of observations, we opted for sparsity-inducing models. In particular, the BART model with and without variable selection was compared to Bayesian linear regression with a horseshoe prior [58], random forest [49], and gradient boosting [59].

Within BART, variable selection was performed using the procedure described by Bleich et al. [60]. This strategy makes use of the “inclusion proportions” of each predictor. Within each posterior sample produced by BART, we can compute the proportion of times that a split using a particular covariate as a splitting variable happens. The variable inclusion proportion of each covariate is then estimated through the posterior mean of the proportions across all of the posterior samples. Being able to calculate this quantity, the selection of variables is carried out as follows:

1. The inclusion proportions of all variables in the model are calculated.
2. The response vector is randomly permuted, thus breaking the relationship between the covariates and the response. The model is retrained with this new response vector, and the variable inclusion proportions are recalculated. These are considered as the existing inclusion proportions in case there is no association between the variables and the response. This step is repeated several times to create a distribution of inclusion proportions that we call the null distribution. This distribution will allow us to see whether the value of the proportion of inclusion of each observed variable is too high with respect to the expected distribution in the event that there is no association. If so, the variable in question would be considered important.
3. In particular, Bleich et al. [60] described three variable inclusion rules described therein. Out of these three rules, the one with the lowest mean squared error was selected using cross-validation.

#### 2.4.1. Evaluation of New Molecules

Given a new candidate molecule described by features *x*, the Bayesian model produces samples from the posterior predictive dispersancy, denoted as p(y|x). The metrics that we utilize to evaluate this molecule are defined as follows:

- Mean predictive dispersancy: the expected value of the posterior predictive distribution defined as E(y|x)=∫y·p(y|x)dy;
- Predictive standard deviation: the standard deviation of the posterior predictive distribution defined as $\sqrt{\int {\left[y-E(y|x)\right]}^{2}\cdot p\left(y|x\right)\;dy}$;
- The expected improvement: if we denote the best dispersancy value observed within the training set as $y^{*}$, the expected improvement of a candidate molecule with features *x* is defined as $\int \max\left(y-y^{*},0\right)\cdot p\left(y|x\right)\;dy$. As argued in [61], expected improvement balances exploration and exploitation and is our chosen metric to decide which targets will end up being synthesized;
- Probability of improvement: the probability that a candidate molecule with features *x* has a dispersancy value higher than $y^{*}$ that is $\int I\left(y>y^{*}\right)\cdot p\left(y|x\right)\;dy$, where I is the indicator function.

All of these metrics can be easily approximated through Monte Carlo using samples from the posterior predictive distribution provided by the BART model.

Finally, to gain some interpretability, BART is used to compute partial dependence plots. These plots serve to illustrate how each of the descriptors affects dispersancy, on average. Additionally, to provide an interpretation of the features obtained from SMILES embeddings, molecular descriptors highly correlated with each of the embedding-based variables are highlighted.

### 2.5. Applicability Domain

An important aspect of predictive modeling is determining the reliability of predictions [62,63]. In this sense, the Applicability Domain (AD) of a model is defined by the chemical subspace of the compounds on which the model has been trained and where the predictions are expected to be accurate [63,64]. Thus, for a new compound, we can determine the prediction reliability by evaluating its AD. In this section, we explain how it is computed for the modeling approaches defined in our tool. The proposed AD metric (Equation (2)) measures the ratio between the average distance of the *k* neighboring molecules to the new candidate (avgDist(X)) and the average distance of each of the *m* molecules in the dataset and their *k* neighboring molecules (${\mathrm{avgDistNeigh}}_{DB}$). A value of 1 for the AD means that the candidate compound has a *k*-nearest neighbor distance that is comparable to the average *k*-nearest neighbor distance for the rest of the molecules in the dataset. Any value lower (greater) than 1 means that the candidate compound is closer (farther apart) to the neighboring compounds than the rest of the compounds in the dataset. The equations are formalized as follows:

(2) $$\begin{array}{cc} \mathrm{AD}(x) & =\frac{\mathrm{avgDist}(x)}{{\mathrm{avgDistNeigh}}_{DB}}\\ \mathrm{avgDist}(x) & =\frac{\sum_{i=1}^{k}d_{i}}{k}\\ {\mathrm{avgDistNeigh}}_{DB} & =\frac{\sum_{j=1}^{m}\mathrm{avgDist}\left(x_{j}\right)}{m}, \end{array}$$

where *x* is the candidate molecule being screened, $d_{i}$ is the distance between the candidate *x* and its neighbor *i*, and $x_{j}$ is each of the *m* molecules in the dataset.

### 2.6. Design of the Visual Analytics Tool

The two machine learning methods described in the previous sections need to be made accessible to the chemical expert. In this regard, a visual analytics strategy is proposed. Visual analytics [65] is a discipline that enables lay users to explore data and to use complex AI methods by means of interactive visualizations.

**Figure 1:**
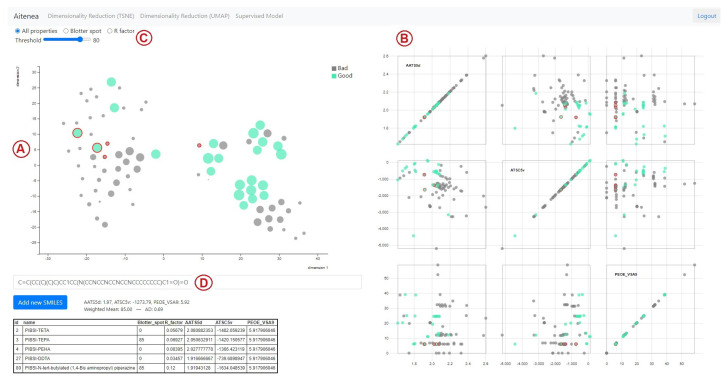
Interface for the model based on structural similarity distances using t-SNE. (**A**). 2D projection of our compounds. (**B**). Scatterplot matrix. (**C**). Filtering of compounds. (**D**). Neighborhood for new compounds. The nodes in green represent the compounds with ‘high’ blotter spot values, i.e., those above the predefined threshold (set as 80 in this plot). The five most structurally similar compounds to the candidate compound are highlighted using a red outline, and their information is summarized in the table included below the t-SNE plot. On the right, scatterplots for pairwise analysis of descriptors are shown, where the descriptor values of the five nearest-neighbors of the candidate are highlighted using a red outline.

We designed two main interfaces that correspond to each of the modeling approaches. The first interface is the frontend for the similarity-based analysis method, and it is shown in Figure 1. This interface facilitates the visual analysis of the entire dataset, and it also allows for adding novel compounds to be analyzed with respect to the other compounds in the dataset. On the left-hand side (Figure 1A), a scatterplot with a two-dimensional projection of the compounds of our dataset is shown. This is achieved by means of the dimensionality reduction techniques t-Distributed Stochastic Neighbor Embedding (tSNE) [66] and Uniform Manifold Approximation and Projection (UMAP) [67]. Both techniques measure distances between data observations in a high-dimensional space and transform those distances to points in a low-dimensional space, usually two-dimensional, so that low-dimensional pairwise distances are related to the distances in the original space.

The main difference between these methods is in the way that distances are computed. tSNE is an algorithm designed for the visualization of high-dimensional data. It first constructs a probability distribution (Gaussian) over the pairs of samples in the original space, in such a way that similar samples are assigned a high probability, while very different samples are assigned low probability. Similarly, the method projects the points from the high dimensional space to a low dimensional space and constructs a Student’s *t*-distribution over pairs of samples in the reduced space. These last two steps are iterated until both spaces, the original and the reduced one, present similar distributions. On the other hand, UMAP is a dimensionality reduction algorithm that builds topological representations of the data both in the original dimension and in the reduced dimension. In the case of high-dimensional data, the topological structure seeks to approximate the manifold on which, it is assumed, the data are located. The topological representation of the low-dimensional data is initialized with random values. Once the topological representations of the data in high and low dimensions have been constructed, the position of the points in the low dimension is optimized to minimize the cross entropy between both topological representations. The projected spaces were obtained using Python’s sklearn.manifold and umap packages that provide implementation of the t-SNE and UMAP methods, respectively [68,69]. For both cases, the default parameters were used, except for t-SNE’s perplexity, which was set to 18 to compensate for the small data context. This parameter is related to the trade-off between the local and global preservation of distances in the data. In this sense, to set the perplexity value, experiments were carried out with different values ranging from 5 to 30, selecting one that showed a good cluster structure.

In the scatterplots generated by these projections, dots correspond to compounds and their size is proportional to the blotter spot value. The location of the dots depends on the dimensionality reduction method used for projecting from the original dimensional space defined by the molecular descriptor values onto a two-dimensional space. For this projection, the tool allows for using either t-SNE or UMAP. When a dot is hovered over, contextual information is shown such as name, dispersancy score (as Blotter spot or R factor), and a visualization of the chemical structure of the compound using SMILES Drawer [70].

On the right-hand side of the interface (Figure 1B), the compounds are visualized using a scatterplot matrix [71], which shows all pairwise combinations of the original molecular descriptor values so that all bivariate relationships between descriptors can be analyzed. These two latter visualizations are coordinated [72], which is a well-known visual information resource to explore data from different perspectives. This is accomplished by brushing and selecting compounds from the main scatterplot, which grays out the compounds on the scatterplot matrix. In this way, the chemical expert can focus the bivariate analysis on a subset of compounds chosen by the user. At the top (Figure 1C), there are some filtering options that toggle visibility for compounds that have: blotter spot values defined only, R factor values defined only, or all compounds. There is also an interactive filter that enables the highlighting of compounds above a certain dispersancy value. All compounds with blotter spot values below the chosen threshold are grayed out.

Finally, it is also possible to add unseen compounds that are intended to be screened and evaluated in a visual manner (Figure 1D). Once a new SMILES code is entered, it is possible to identify the nearest neighbors of the dataset as detailed in Section 2.3.2. The nearest neighbors are highlighted—in the main scatterplot and in the scatterplot matrix—using a red outline. A tabular view of the neighbors with their descriptor values is shown at the bottom, while the predicted value and applicability domain estimate based on the similarity-based analysis are displayed just above the table.

**Figure 2:**
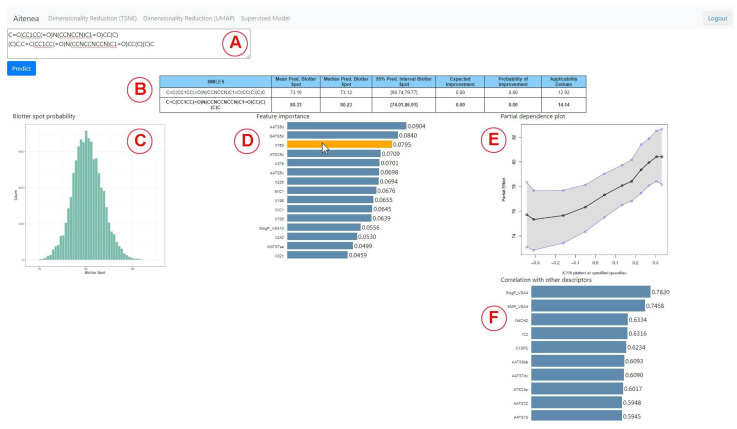
Interface for the model using BART. The table summarizes the performance of the entered molecules (**A**,**B**). The histogram shows the posterior probability for the compound clicked from the table (**C**). Feature importance, a partial dependency plot and correlation of a descriptor with other descriptors are also shown (**D**–**F**). For instance, we can see in the partial dependence plot that the Blotter spot value tends to increase for larger values of the selected variable (the embedding X758), while the correlation bar chart shows that this embedding correlates highly with the descriptor SlogP_VSA4.

On a different tab, the interface enables the chemical expert to use the BART method described in Section 2.4; in addition, it sheds light on the inner workings and the impact of each descriptor. Once a sequence of comma-separated SMILES codes is entered in the main text area (Figure 2A), and the *Predict* button is clicked, the molecular descriptors and molecular embeddings described in Section 2.2 are computed for those chemicals. Once the descriptors and embeddings are computed, then BART is used to predict the target distribution of the Blotter spot value for the entered compounds. This information is summarized with a table (Figure 2B) that contains for each row:

- SMILES of the compound for prediction;
- Mean value of the posterior predictive distribution as described in Section 2.4.1;
- Median value of the posterior predictive distribution;
- 95% prediction interval as described in Section 2.4.1;
- Expected improvement of the predicted blotter spot value with regard to the highest blotter spot of the training dataset as described in Section 2.4.1;
- Probability of improvement with respect to the highest blotter spot of the training dataset as described in Section 2.4.1;
- Applicability domain measured as described in Section 2.5.

**Figure 3:**
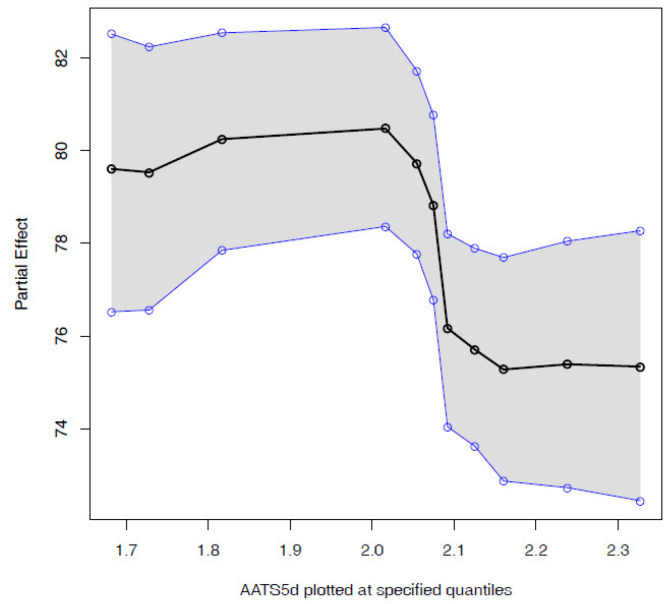
Partial dependence plot for AATS5d. This plot facilitates the understanding of how the dispersancy value is affected as a given descriptor is modified.

The user can interact with any of the predicted compounds by clicking on the corresponding row to render two new graphical elements, i.e, a histogram (Figure 2C) and a bar plot (Figure 2D). The histogram shows a sample of the posterior distribution for the predicted blotter spot value. The bar plot shows the importance of each feature—either molecular descriptor or molecular embedding—as described in Section 2.4.1. The chemical expert can gain insights on the contribution of each feature by two additional plots. The first one is the *Partial dependence plot* (PDP), which shows for any chosen feature how the dispersancy value changes on average (black line) at different deciles of the feature value, marginalizing over the values of the rest of the features. Similarly, the plot also displays 95% percent intervals at different deciles (blue lines). For instance, for the molecular descriptor AATS5d (Figure 3), values of this feature ranging in the [0, 0.3] percentile ([1.7, 2.0] in the AATS5d descriptor) show an average blotter spot value of around 80, while values of this feature in the [0.6, 1] percentile ([2.1, 2.3] in the AATS5d descriptor) generate dispersancy blotter spot values around 75.

Although the molecular embeddings were shown to be useful for prediction, they lack interpretability. Therefore, we aimed at incorporating a bar plot that ranks the top 10 molecular descriptors that best correlate with the chosen molecular embedding (Figure 2F). For instance, for the molecular embedding X758, we notice that the molecular descriptor SlogP_SA4, which is based on Wildman–Crippen LogP value, highly correlates with this descriptor. This allows for determining that this molecular embedding encodes information related to the hydrophobicity of the compound. A short video highlighting the main functionalities of the tool can be found in the Supplementary Material section.

## 3. Results and Discussion

This section presents the results corresponding to the statistical validation of the predictive models included in our tool and discusses, through the resolution of a use case, how it can be applied in a virtual screening problem.

### 3.1. Evaluation of the Models

We performed an evaluation of our models through cross validation [73,74]. The performance metrics used for this purpose are Mean Absolute Error (MAE) and Root Mean Squared Error (RMSE). The structural similarity-based model was statistically evaluated via 5-fold cross-validation. As discussed in Section 2.3.2, two methods, namely Kmeans and DBSCAN, were used to split the data by structural diversity in order to propose an evaluation consistent with the approach of the proposed model. In this regard, once the *k* most similar molecular structures were obtained in terms of the descriptor space, the Weighted Mean (WM) was calculated for each compound in the test set. The metric used to measure the distances between the molecular structures was the normalized Euclidean distances distance, and the number of neighbors was set to five (k=5). The performance obtained is reported in Table 2.

For the Bayesian regression model, RMSE and MAE were estimated via repeated 5-fold cross-validation with 10 repetitions. Unlike the evaluation of the model based on structural similarity, here we choose to do a random data splitting given the higher number of covariates that are present in this model. Table 3 shows the results obtained. The best performing model was Random Forest. However, its performance is comparable with that of Gradient Boosting and BART with feature selection. As discussed in Section 2.4, Bayesian approaches provide probabilistic forecasts that allow for quantifying uncertainty in the predictions, which is of paramount importance in our application domain. This is the reason we selected BART with variable selection as the final model, albeit its slightly lower performance compared to that of Random Forest and Gradient Boosting.

### 3.2. Virtual Screening Using the Visual Analytics Tool

The models used for predicting blotter spot effectively allow for capturing interesting patterns within the dataset. However, to achieve high prediction performance, interpretability is compromised. There are two sources that preclude any possible form of rationalization of the criteria used by the algorithm: the black-box nature of the models used and the abstract nature of the molecular descriptors (both the traditional ones and the embeddings). To help explaining the inner workings of the model, we can use partial dependence plots (PDPs), as the ones in Figure 3. PDPs allow for quantifying the dependency between the predicted blotter spot and a set of descriptors of interest, marginalizing over the values of all other descriptors. However, this does not help with the fact that some molecular descriptors are still too abstract to provide us with a useful understanding about the decisions made by the model.

**Figure 4:**
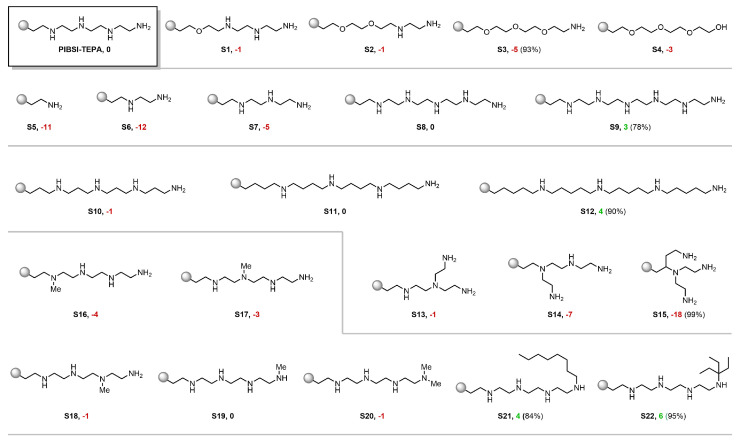
Virtual screening of PIBSI-TEPA derivatives. The values following the product number indicate the difference between the mean posterior predictive blotter dispersancy and PIBSI-TEPA’s experimental value used for training (85%). The percentage in parenthesis expresses the certainty of the Bayesian model for placing the prediction above (for cases in which the predictive mean is above 85%, represented in green) or below (for cases in which the predictive mean is below 85%, represented in red) the PIBSI-TEPA reference.

In an attempt to further examine the predictive potential of our model and understand its inner workings, we proceeded to evaluate a series of virtual PIBSI molecules derived from a known reference substrate. In particular, PIBSI-TEPA was selected as a starting point for its simplicity and used to design similar derivatives which might reveal logical trends in the predictions. For that purpose, we computed the mean posterior predictive blotter spot of each derivative and compare it with that of PIBSI-TEPA (which was obtained experimentally and has value $y_{PT}=85\%$). In addition, to give a measure of strength of the tendencies that may arise, we computed the posterior probability of the derivative having a higher (lower) dispersancy than the PIBSI-TEPA that is $\int I\left(y≷y_{PT}\right)\cdot p\left(y|x\right)\;\;dy$. This integral was approximated through Monte Carlo using the sample from the posterior predictive distribution provided by the BART model. The results are illustrated in Figure 4.

First, a cumulative replacement of the amino groups in the PIBSI-TEPA by oxygen caused the predicted blotter spot dispersancy to decrease down to a minimum when all the internal amines were removed (Figure 4, S1–S3). The stark influence of basic amino groups over other common heteroatoms has been consistently observed throughout the literature [7,9]. Therefore, this result stands in agreement with well-known empirical trends. The slight recovery in the dispersant activity when replacing the terminal nitrogen by an OH group could be interpreted as the acidic proton offsetting the loss of NH2 (Figure 4, S4).

Along these lines, the groups of Mekewi and Abdel Azim [28,29,38] studied homologue series of designer PIBSI dispersants varying in their number of ethyleneamino subunits. The studies revealed a consistently high degree of correlation between the number of amino monomers and the blotter spot value. This tendency was also accurately captured by our model as we probed a series of PIBSI-TEPA homologues. Polyethyeleneamino chains shorter than TEPA proved to be less effective than the reference substrate with a difference of up to 12 percentage points in blotter spot (Figure 4, S5–S7). On the contrary, a penta-ethyeleneamino scaffold did not lead to any appreciable change in the predicted dispersancy, while further attaching an additional monomer resulted in a slight improvement (Figure 4, S8–S9). This shows that the model computes amino groups as a positive contribution altogether, and suggests that the activity will further increase proportionally with more amines integrated into the polar head. Nonetheless, it is worth mentioning that our current model has not been trained with limiting factors such as the detrimental effect of nitrogen-rich compounds in the seal performance of elastomers. Therefore, too optimistic predictions for large polyamine moieties should be considered with caution.

The conformational flexibility of the polar head for maximizing the interaction with the randomly distributed acidic binding sites on the surface of soot particles has been proposed to be another decisive factor for a high particle-dispersant affinity [7]. In practice, this implies that polar heads with a long and linear configuration are expected to have a higher dispersancy efficiency compared to their branched analogs. To examine the performance of our model in this regard, on the one hand, stretched TEPA derivatives were tested by inserting additional methylene groups to each repeating subunit (Figure 4, S10–S12). Interestingly, a positive response was observed when the chain length was sufficiently extended. On the other hand, as the ethyleneamino units of the TEPA were rearranged to create branches, the predictions entailed severe penalties as the ramification increases (Figure 4, S13–S15). Based on this analysis, it appears that the algorithm has coherently captured the essence of this mechanistic aspect.

Finally, encouraged by the consistency of the predictions with known experimental observations, we further explored subtle structural changes to obtain information towards designing an improved dispersant candidate. For instance, we probed the influence of each NH group from PIBSI-TEPA by simulating selective N-methylations. This revealed a higher sensitivity of NH groups towards the loss of dispersancy the closer they are to the succinimide linkage (Figure 4, S16–S19). At the terminal NH2 site, a decrease in the activity was only observed when converted into a tertiary amine (Figure 4, S20). The low impact of mono-alkylation at this site could then be exploited by introducing a large hydrophobic group, known to benefit dispersancy [75], and we, consequently, expected our model to have learned this feature. Indeed, both a long hydrocarbon chain and a bulky tertiary alkyl fragment led to a notable increase in the predicted blotter spot (Figure 4, S21–S22). Structures such as the former are of special interest, since it can be prepared through a presumably simple synthetic process using available feedstock chemicals. As additional information, a view of compound S21 evaluated with the structural similarity-based model using t-SNE can be seen in Figure 1. The model obtained a predicted value of 85 with an AD of 0.69. Information of the five most similar compounds to S21 in the database, including PIBSI-TEPA, is summarized in the table below the t-SNE plot.

## 4. Conclusions

The use of AI is having a growing impact on the design of new molecular compounds. Although it does not replace some of the traditional wet-lab experimentation steps, it is playing a key role in accelerating discovery of new materials such as dispersants. In this work, we have illustrated how a structural similarity-based model and a Bayesian regression model can be successfully combined for the virtual screening of dispersants in the challenging context of a small data problem. We present a dataset of 53 potential dispersants constructed from the literature and design a variety of models to predict the dispersancy efficiency property. In addition, a visual analytics tool for exploring interactively the data and models has been presented. This tool aims to facilitate the involvement of chemical experts with the outputs produced by the machine learning models, contributing in this way to the interpretability of the results. Finally, a brief study was presented to illustrate the analysis that a chemical expert can follow with the tool to perform virtual screening of a molecular structure. In this sense, the performance of our models was evaluated with a virtual screening of representative polar head designs, which revealed a high consistency with well-known empirical trends in the blotter spot predictions. In summary, our AI methodology would provide useful insights to material designers beyond the limits of a classical Edisonian approach to materials discovery.

## Figures and Tables

**Table 1:** Four subsets of descriptors selected for the model based on structural similarity.

Subset	Feature Selection Method	Pre-Selected Molecular Descriptor
1	Random Forest	AATS5d, AATS5Z, SIC0, ATSC3d, ATSC5v, GATS5d,
		AATS5v, GATS3d
2	Decision Tree	AATS5d, ATSC5v, ATSC6Z, AATS8p, AATSC7d,
		MINssCH2, PEOE_VSA9, GATS8i
3	Decision Tree	AATS5d, ATSC5v, GATS8p, GATS8v, GATS1se,
		MINssCH2, AATS1Z, GATS8i
4	Expert Criterion	nBase, nN, MATS1se, AATSC0p, TopoPSA(NO)

**Table 2:** MAE and RMSE for k-nearest neighbors broken down by the clustering method used for sampling the folds.

Data Sampling	MAE	RMSE
KMeans	3.89±1.21	5.45±1.65
DBSCAN	3.84±1.19	5.37±1.78

**Table 3:** MAE and RSME of different models estimated through repeated 5-fold cross validation.

Algorithm	MAE	RMSE
Bayesian Regression + horseshoe prior	6.38±0.51	8.34±0.65
BART	6.02±0.36	7.61±0.38
BART + Variable Selection	5.50±0.34	7.56±0.47
Random Forest	5.10±0.26	6.78±0.17
Gradient Boosting	5.34±0.39	7.11±0.52

## Data Availability

The dataset can be found in our GitHub repository: https://github.com/jimenamartinez/Dispersancy_Efficiency (accessed on 14 February 2023).

## Supplementary Materials

The following supporting information can be downloaded at: https://www.mdpi.com/article/10.3390/polym15051324/s1, Video S1: Visual Analytics Tool for Dispersancy Efficiency.
